# Characterizing the fecal microbiome in patients on the ketogenic diet for drug resistant epilepsy

**DOI:** 10.1016/j.heliyon.2025.e41631

**Published:** 2025-01-02

**Authors:** Alexander Freibauer, Nikhil Pai, Rajesh RamachandranNair

**Affiliations:** a McMaster University, Department of Pediatrics, Hamilton, ON, Canada; b McMaster Children's Hospital, Division of Pediatric Neurology, Hamilton, ON, Canada; c McMaster Children's Hospital, Division of Pediatric Gastroenterology, Hepatology and Nutrition, Hamilton, ON, Canada; d University of Pennsylvania, Perelman School of Medicine, Department of Pediatrics, Philadelphia, PA, USA; e Children's Hospital of Philadelphia, Department of Gastroenterology, Hepatology & Nutrition, Philadelphia, PA, USA

## Abstract

**Background:**

The ketogenic diet is a dietary therapy with anti-seizure effects. The efficacy of the diet is variable, with initial animal studies suggesting the intestinal microbiome may have a modulating effect. Initial research on the role of the human microbiome in pediatric epilepsy management has been inconclusive.

**Methods:**

In this single-center prospective cohort study, stool samples were collected from 4 patients with drug resistant epilepsy on the ketogenic diet and 9 with drug resistant epilepsy as controls. The samples were analyzed by 16S RNA sequencing.

**Results:**

A trend towards increased alpha diversity was noted among patients on the ketogenic diet compared to the control group. Patients on the ketogenic diet also trended towards a higher relative abundance of *Bacteroidaceae, Ruminococcaceae*, and *Prevotellaceae* species. A subset of the control group had a high relative abundance of *Bifidobacterium*, which may make them a candidate for a trial of the ketogenic diet as a therapeutic option.

**Conclusion:**

These findings add to the growing field of research of how the ketogenic diet modulates the intestinal microbiome in pediatric epilepsy patients. Future emphasis on multi-centre trials, consistent stool collection practices and the establishment of standardized stool biobanking protocols are needed further to validate these novel findings in a pediatric population.

## 1 Introduction

The ketogenic diet (KD) is a low carbohydrate, high-fat diet with anti-seizure effects. The efficacy of the ketogenic diet is quite variable, with only 40–50 % of patients achieving >50 % seizure reduction on the diet [1,2]. Even in patients who can achieve seizure reduction on the diet, this often comes at the cost of significant paternal stress [3]. Continued work is needed to establish biomarkers within the microbiome to help predict whether a patient will respond to the ketogenic diet and identify if there is enrichment of specific bacteria that may be associated with seizure reduction [4]. This may include an understanding of both the compositional, and functional changes in the microbiome that confer response. While the mechanisms underlying the anti-seizure properties of the KD are unclear, inhibition via poly-unsaturated fatty acids and GABA have been theorized [5]. Another potential mechanism underlying the ketogenic diet's efficacy is the intestinal microbiome's influence [6]. Diet is a key mediator of gut microbial composition, and alterations in the gut microbiome have been associated with obesity, inflammatory bowel diseases, and epilepsy [7,8]. Extensive evidence from animal models and clinical studies also suggests a strong brain-gut connection, whereby intestinal microbial changes, directly and indirectly, influence neurological and psychiatric disease. Animal studies have directly suggested that the anti-seizure effects of the ketogenic diet may be mediated by the fecal microbiome [9].

Clinical research on the interactions between the ketogenic diet and the microbiome has been limited. A recent paper published by Dahlin et al. where 28 patients with drug-refractory epilepsy were treated with the ketogenic diet, found that the presence of specific bacterial taxa (*Gordonibacter pamelaeae, Eggerthella lentha,* and *Bifidobacterium longum susp. longum)* were associated with response to KD vs non-response. A limited number of studies have assessed gut microbial changes with KD treatment in pediatric epilepsy [[10], [11], [12], [13], [14], [15], [16]]. Due to small sample sizes, variability in the measurement of the fecal microbiome and differences in the duration of KD, comparison between studies is difficult. No clear consensus exists towards the effects of the KD on the intestinal microbiome [[10], [11], [12], [13], [14], [15], [16]].

In this single-centre prospective cohort study, we describe changes in the intestinal microbiome of pediatric patients receiving KD and microbiome-based predictors of KD response. We also describe strategies for ongoing research in this area to strengthen the discovery of microbial biomarkers of response, including the feasibility of measuring microbial changes in future pediatric drug-resistant epilepsy patients treated with KD.

## 2 Methods

We performed a single-centre cross-sectional study of children with drug resistant epilepsy (DRE) at McMaster Children's Hospital (Hamilton, Canada). DRE was defined as “failure of adequate trials of two tolerated and appropriately chosen and used anti-seizure medication schedules (whether as monotherapies or in combination) to achieve sustained seizure freedom.” Patients were between 1 and 18 years of age and were followed through the Division of Pediatric Neurology at McMaster Children's Hospital. Patients were selected if they met relevant clinical criteria across one of four anticipated cohorts: 1) patients with DRE not on KD 2) patients with DRE on KD with poor seizure control (<50 % reduction in seizure frequency), 3) Patients with DRE on low ratio KD with good seizure control (>50 % reduction in seizure frequency) and 4) patients with DRE on high ratio KD with good seizure control. Patients on KD were followed by a multidisciplinary team including a pediatric neurologist, dietician, and nursing support. Patients on KD had to have been on diet for >3 months. Patients were excluded if they had received an antibiotic within 3 months of the inclusion of the study. This study received full institutional ethics board approval by the Hamilton Integrated Research Ethics Board (Project Number 8224) on July 21, 2020, and all patients and/or substitute decision-makers provided written informed consent.

For sample collection, the patient or substitute decision-maker was shipped a sample collection kit, including a sterile scoop and cryogenic tube. Within 48 h of sample collection, a study team member would pick up the sample from the patient's residence. All samples were stored in patients' home freezers prior to collection and then stored at −80 °C upon labeling. All patient data was collected retrospectively.

We projected a sample size 40 with 10 in each subgroup, which was chosen based on institutional capacity for recruitment for the purpose of assessing the feasibility of recruitment. All samples were analyzed for gut microbial composition by 16S RNA sequencing, and a biostatistician translated sequencing results into figures that could be interpreted for underlying trends.

## 3 Results

**Fig. 1:**
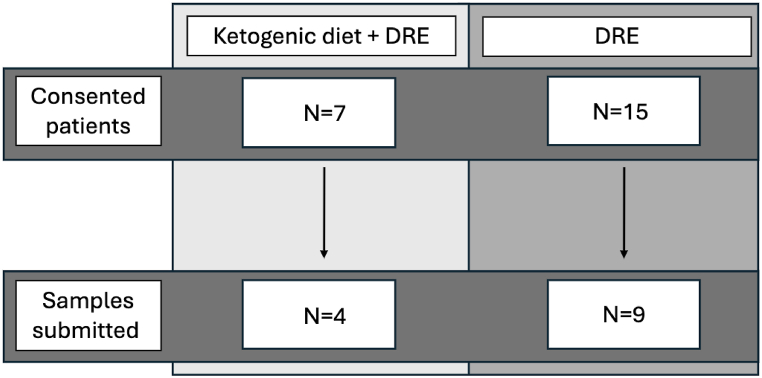
Flow chart showing patients who consented to participate in the study compared to those who submitted samples for analysis.

Between November 2021 and December 2023, 22 patients consented to participate in the study, 7 with KD and 15 with medically refractory epilepsy (Table 1). 4/7 patients on KD and 9/15 patients with medically refractory epilepsy submitted a stool sample (Fig. 1). Among patients on KD, two patients had a 3:1 ratio (lipids:protein/carbohydrates), and two had a 4:1 ratio. As our sample size was small, we did not feel that comparisons between the microbiota of low vs. high KD-ratio would offer a meaningful comparison. We do believe this is a meaningful clinical question that should be answered in follow-up studies. Included were on a KD for an average of 53.5 months (12–90 months).

**Table 1:** Demographics of patients who submitted a sample for fecal microbiome analysis.

	Ketogenic diet	Medically Refractory Epilepsy
**Number of patients**	4	9
**Age (y)**	9.19	6.38
**Age (y) at first seizure**	2.01	2.05
**Average seizure frequency (daily)**	33	33
**Etiology**		
Structural	1	3
Metabolic	1	0
Genetic	2	6
Unknown	0	0
**Average number of seizure types**	3.25	3.11
**Average number of ASM**	0.75	2.56
**Developmental delay**	4/4	9/9
**Autism**	1/4	3/9
**Motor function**		
Ambulatory without assistance	3	3
Ambulatory with assistance	0	2
Non-ambulatory	1	4

13 patients were enrolled across all four study arms. The study did not meet the intended recruitment goals.

**Fig. 2:**
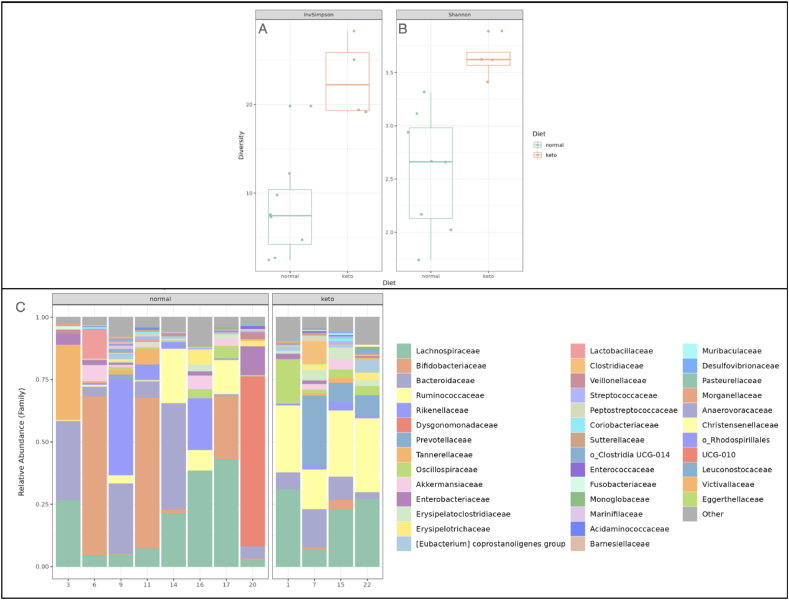
16S Microbiome Sequencing data. Alpha diversity of the cross-sectional cohort of medically refractory patients on the ketogenic diet, or regular diet as demonstrated as a box plot, with each dot representing an individual patient as measured by the inverted Simpson index (A), or Shannon index (B). Relative abundances of bacterial families were represented in a stacked bar graph, with each bar representing an individual patient, and each colour corresponding to a bacterial family (C).

16S rRNA data was collected for each patient. A trend towards increased alpha diversity was noted among patients on the ketogenic diet compared to the intended control group of patients with DRE, as shown via the Inverse Simpson index in Fig. 2A, and Shannon index in Fig. 2B. Relative abundance of taxa was also shown for each patient, as demonstrated in Fig. 2C. Patients on the ketogenic diet trended towards a higher relative abundance of *Bacteroidaceae, Ruminococcaceae*, and *Prevotellaceae* species. Among the DRE group, a subset of patients had increased relative abundance of *Bifidobacteriaceae*, *Rikenellaceae*, *Tannerellaceae*, and *Dysgonomonadaceae* (Fig. 2C).

## 4 Discussion

This study adds to the limited literature describing changes in the pediatric gut microbiome among patients with DRE on the KD. While conclusions may be strengthened in future studies, we identified several trends of interest. We showed a trend towards increased diversity in our pediatric ketogenic diet cohort, which is inconsistent with prior data in the literature. Increased microbial diversity among patients on KD in our study may be attributed to various factors, such as patient age, geographic location (urban versus rural), dietary changes at the time of sampling, underlying etiology of DRE, and KD duration. The complex interplay of these confounders is beyond the scope of our sample size and would require further study to clarify. Nonetheless, our findings contribute to a growing body of heterogeneous clinical data, offering a basis for future meta-analyses.

Our observation of elevated alpha diversity within the KD group suggests that while alpha diversity captures broad shifts in microbiome composition, it does not account for specific changes in microbial taxa or functional capacity. Future research might better identify distinct microbial signatures associated with KD beyond diversity metrics alone. However, alpha and beta diversity remain widely used indices to describe broad shifts in the gut microbiome [3,4,17]. Additionally, our findings align with prior studies in both adults and children on KD, including our observed increased relative abundance of *Bacteroides* and *Prevotellaceae* species in patients with epilepsy on KD [[10], [11], [12]].

We also found a relative enrichment of Bifidobacterium among three patients in our medically refractory epilepsy cohort. Notably, prior reports suggest the presence of Bifidobacterium in the medically refractory epilepsy patients population conveys the potential efficacy of the ketogenic diet [14]. The mechanism of this benefit is thought to be due to the reduction of proinflammatory effects of Bifidobacterium, which cannot survive in the microbiome of patients on the KD [14]. While these patients served as a control in our study and did not receive KD, further studies research may be performed would need to assess whether Bifidobacterium may serve a prognostic value for predicting potential benefit from the trial of the ketogenic diet.

In this single-centre prospective study, recruitment was conducted over two years. We could not recruit sufficient patients to meet our target of 40 patients, with only 4 samples collected from patients on the ketogenic diet. Several factors may have contributed to these recruitment challenges, which are important to consider in designing and implementing future pediatric studies where dietary management of epilepsy typically has greater uptake by willing patients and families.

Only 57 % of recruited participants submitted stool samples despite steps taken to mitigate the burdens of stool collection on families (mailing collection kits to patients’ homes, couriering samples directly from their homes). Stool collection is typically seen as burdensome for patients and providers, and may be a deterrent to participation, particularly in a patient cohort with the simultaneous complexities of administering a ketogenic diet. Financial incentives may help offset these challenges by increasing the value of research participation for study subjects. Finally, the impacts of the COVID-19 pandemic hindered recruitment efforts, given the innumerable challenges of patient care and research conduct during this time. The changing nature of clinical care from in-person to virtual visits may have also contributed to reduced recruitment during this time and hereafter.

Our data and the challenges experienced with recruitment highlight why a single-centre prospective study of the microbiome in pediatric epilepsy patients will not attract the sample sizes required to validate these findings further. Our experience is supported by other single-centre studies with similar limited recruitment. 7 prior single center studies recruited only between 6 and 28 patients on the ketogenic diet [[10], [11], [12], [13], [14], [15], [16]]. The study with the greatest recruitment of 28 patients [12] required extended recruitment over at least 3 years, based on an initial cohort of 12 patients reported in 2019 [13].

Establishment of establishing multi-centre trials, with centralized analysis and reporting, would significantly enhance data collection and interpretation. Systematic approaches to biobanking stool among patients receiving KD may significantly enhance recruitment. This approach has been useful across other pediatric disorders, including pediatric inflammatory bowel disease, to generate large, untargeted datasets to support ongoing studies [[18], [19], [20]]. The relative rarity of DRE treated with the KD across Canada and the network of practitioners across the country suggest non-profit or foundation-based funding may support the establishment of national biobanking protocols to enhance future studies.

## 5 Conclusion

The intestinal microbiome remains an area of interest in identifying gaps in knowledge of the mechanism of medically refractory epilepsy and the ketogenic diet in the pediatric population. Our study identified a novel trend of increased alpha diversity of patients on the ketogenic diet and identified patients with medically refractory epilepsy with a high relative abundance of Bifidobacterium, which may make them a candidate for a trial of the ketogenic diet as a therapeutic option. Future emphasis on multi-centre trials, consistent stool collection practices and the establishment of standardized stool biobanking protocols are needed to validate these novel findings in a pediatric population.

## CRediT authorship contribution statement

**Alexander Freibauer:** Writing – original draft, Resources, Project administration, Methodology, Investigation, Data curation, Conceptualization. **Nikhil Pai:** Writing – review & editing, Supervision, Resources, Methodology, Formal analysis. **Rajesh RamachandranNair:** Writing – review & editing, Supervision, Resources, Formal analysis, Conceptualization.

## Declaration of competing interest

The authors declare that they have no known competing financial interests or personal relationships that could have appeared to influence the work reported in this paper.
